# Psychogenic Rheumatism: Its Differentiation from True Rheumatic Disease
*Abstract of an address to the Bath, Bristol and Somerset Branch of the B.M.A., at Bath, October 19th, 1948, and *Postgraduate Medicine,* 1947, I, VI.


**Published:** 1949-04

**Authors:** Philip S. Hench

**Affiliations:** Professor of Medicine, University of Minnesota (Mayo Foundation). Head of a section in the Division of Medicine, Mayo Clinic, Rochester, Minnesota, U.S.A.


### PSYCHOGENIC RHEUMATISM: ITS DIFFERENTIATION FROM TRUE RHEUMATIC DISEASE *

BY

PHILIP S. HENCH, M.D.

*Professor of Medicine, University of Minnesota (Mayo Foundation).*
*Head of a section in the Division of Medicine, Mayo Clinic,*
*Rochester, Minnesota, U.S.A.*

---

TWO of the many conditions commonly called " rheumatism" are actual joint diseases—(1) osteo-arthritis, a nuisance but not a calamity, and (2) rheumatoid arthritis, the most crippling of all. In two others there is no joint disease: (3) primary fibrositis, a periarticular or intramuscular condition producing aching and stiffness, and (4) " psychogenic rheumatism "—a psychoneurosis affecting the musculo-skeletal system and sometimes confounded with one of the others.

Osteo-arthritis causes aching and stiffness. It commonly affects the hips and lumbar spine, and the hands. There is no constitutional evidence of disease—anæmia, raised sedimentation-rate, etc. The joints are not swollen and movement, though painful, is little restricted : no flexion deformities or ankylosis. The essential feature is senile degeneration of cartilage : this becomes thin, sometimes even to exposure of underlying bone. The bone thickens and hardens—" eburnation," and there may be osteophytic lipping round the joints. The bone changes are hypertrophic rather than atrophic. The synovial membrane is not affected (no hydrops or pannus): the capsule of the joint and the muscles lose resiliency, " secondary fibrositis", without spasm or significant deformities. In X-rays, we see the joint-space narrowed, from absorption of cartilage. The bone margin throws a denser shadow than normal and osteophytes may be seen.

In rheumatoid arthritis there is aching and swelling of the joints: many joints are affected, especially those of the fingers. The fusiform swelling is often characteristic and, in addition, there is evidence of constitutional disease—raised sedimentation-rate and secondary anæmia. The swelling of joints is due chiefly to disease

* Abstract of an address to the Bath, Bristol and Somerset Branch of the B.M.A., at Bath, October 19th, 1948, and *Postgraduate Medicine*, 1947, I, vi.

of the synovial membrane, which becomes thickened and proliferates with formation of villi. Collections of round cells are a striking feature in the synovial membrane, in the adjacent bone and fibrous and muscular tissues. As disease progresses, the affected synovial membrane sends out a tongue, or pannus, of inflammatory tissue: first over, and then into, the cartilage. The cartilage is partly destroyed and the pannus produces adhesions between the joint surfaces, first fibrous then osseous. The inflammation in capsule and muscles gives rise to spasm and flexion deformities. In the X-rays we see periarticular cloudiness due to synovial thickening; atrophy or decalcification of underlying bone and narrowing of joint spaces. The changes in the synovial membrane, signs of systemic disease, muscular atrophy and flexion deformities distinguish this condition from the former. X-ray changes may not be obvious in the early phases.

Primary fibrositis produces characteristic aching pains in muscles of neck, shoulders, chest, etc. No evidence of systemic disease; sedimentation-rate normal; X-rays show nothing to explain symptoms. This is a primary or idiopathic condition: myositis or periarticular fibrosis without joint disease. No definite swelling, no deformities, no limitation of movement: stiffness generally worse in early morning, after rest, or in damp weather—relieved by heat, aspirin or movement. Biopsy shows no synovial changes.

"Psychogenic rheumatism" is a condition characterized by pains in various and many situations, without evidence of organic disease; no, or no material, X-ray changes; normal sedimentation-rate, etc. There may be other evidence of functional disturbance: the patient is introspective and non-co-operative. Moods and feelings may influence symptoms, but usually not temperature and weather; exercise makes symptoms worse, and rest relieves; heat, aspirin and similar treatment do not help.

"Psychogenic rheumatism" is the musculo-skeletal expression of functional disorders, tension states or psychoneuroses, and is one of the common causes of aches and pains. In the Army's rheumatism centres about 15 per cent. of all "rheumatic" patients were psychogenic, generally with no evidence of organic disease. In some cases "psychogenic rheumatism" was a marked functional overlay of some rheumatic disease such as fibrositis. In most, there is only a collection of symptoms. The underlying cause may be combat fatigue—certain soldiers just "couldn't take any more", with resultant "flight into illness". In other cases the basic difficulties were maladjustment to discipline, to criticism, to menial job, to the "indignities" and lack of privacy; to homesickness, to loneliness, to worry over illness or fidelity of wife; or to financial troubles.

In civilian life the basic difficulties may be such as these: a stern parent, an unattractive girl afraid her "chances are going", a veteran forced by housing difficulties to live with " in-laws ", lack of holidays, sexual difficulties, ageing spinsterhood—or even fear of rheumatism ! In fact, different kinds of fears and frustrations. The family doctor, with his knowledge of the patient's personality and circumstances, has a unique opportunity to detect this condition. In " psychogenic rheumatism", " rheumatism remedies " are of little avail, except as a mild form of psychotherapy. These miserable souls need something more than heat, aspirin, etc. The results of psychiatric treatment are often most gratifying ; but the first essential is the recognition by physician and patient that the trouble is the functional condition I have endeavoured to portıay.

---

It seems that the difficulties which confront many country practitioners under the " Health " Act existed also in mediæval Mantua.

> " I do remember an apothecary,
> In tattered weeds, with overwhelming brows,
> Culling of simples ; meagre were his looks,
> Sharp misery had worn him to the bones :
> And in his needy shop . . . about his shelves
> A beggarly account of empty boxes,
> Remnants of pack thread and old cakes of roses
> Were thinly scatter'd to make up a show."
>
> " Art thou so bare and full of wretchedness,
> And fear'st to die ? Famine is in thy cheeks,
> Need and oppression starveth in thine eyes,
> Contempt and beggary hang upon thy back ;
> The world is not thy friend nor the world's law :
> The world affords no law to make thee rich."
>
> *Romeo and Juliet,* Act v, Sc. i.

